# Deficiency of LMP10 Attenuates Diet-Induced Atherosclerosis by Inhibiting Macrophage Polarization and Inflammation in Apolipoprotein E Deficient Mice

**DOI:** 10.3389/fcell.2020.592048

**Published:** 2020-10-23

**Authors:** Jiawei Liao, Xiangbo An, Xiaolei Yang, Qiu-Yue Lin, Shuang Liu, Yunpeng Xie, Jie Bai, Yun-Long Xia, Hui-Hua Li

**Affiliations:** ^1^Department of Cardiology, Institute of Cardiovascular Diseases, The First Affiliated Hospital of Dalian Medical University, Dalian, China; ^2^Department of Interventional Therapy, The First Affiliated Hospital of Dalian Medical University, Dalian, China; ^3^Department of Occupational and Environmental Health, School of Public Health, Dalian Medical University, Dalian, China; ^4^Beijing Key Laboratory of Cardiopulmonary Cerebral Resuscitation, Department of Emergency Medicine, Beijing Chaoyang Hospital, Capital Medical University, Beijing, China

**Keywords:** immunoproteasome, LMP10, diet, atherosclerosis, macrophage, inflammation, polarization

## Abstract

Macrophage polarization and inflammation are key factors for the onset and progression of atherosclerosis. The immunoproteasome complex consists of three inducible catalytic subunits (LMP2, LMP10, and LMP7) that play a critical role in the regulation of these risk factors. We recently demonstrated that the LMP7 subunit promotes diet-induced atherosclerosis via inhibition of MERTK-mediated efferocytosis. Here, we explored the role of another subunit of LMP10 in the disease process, using ApoE knockout (ko) mice fed on an atherogenic diet (ATD) containing 0.5% cholesterol and 20% fat for 8 weeks as an *in vivo* atherosclerosis model. We observed that ATD significantly upregulated LMP10 expression in aortic lesions, which were primarily co-localized with plaque macrophages. Conversely, deletion of LMP10 markedly attenuated atherosclerotic lesion area, CD68^+^ macrophage accumulation, and necrotic core expansion in the plaques, but did not change plasma metabolic parameters, lesional SM22α^+^ smooth muscle cells, or collagen content. Myeloid-specific deletion of LMP10 by bone marrow transplantation resulted in similar phenotypes. Furthermore, deletion of LMP10 remarkably reduced aortic macrophage infiltration and increased M2/M1 ratio, accompanied by decreased expression of pro-inflammatory M1 cytokines (MCP-1, IL-1, and IL-6) and increased expression of anti-inflammatory M2 cytokines (IL-4 and IL-10). In addition, we confirmed in cultured macrophages that LMP10 deletion blunted macrophage polarization and inflammation during ox-LDL-induced foam cell formation *in vitro*, which was associated with decreased IκBα degradation and NF-κB activation. Our results show that the immunoproteasome subunit LMP10 promoted diet-induced atherosclerosis in ApoE ko mice possibly through regulation of NF-κB-mediated macrophage polarization and inflammation. Targeting LMP10 may represent a new therapeutic approach for atherosclerosis.

## Introduction

Atherosclerosis is a chronic immune-inflammatory response within the artery, with lipid accumulation and oxidation in the intima being hallmarks of the disease (Ross, 1999). Once oxidized lipids accumulate in the intima, they attract circulating monocytes to enter the sub-endothelial space and differentiate into macrophages to scavenge these oxidized lipids (Moore et al., 2013). These lipid-laden macrophages (also known as foam cells) then secrete inflammatory cytokines, including chemokines and adhesion molecules, as well as pro- and anti-inflammatory cytokines, to recruit more monocytes, initiating inflammatory responses (Moore et al., 2013). Additionally, accumulation of oxidized lipids in foam cells activates the production of reactive oxygen species that cause cellular oxidative stress, leading to apoptotic and necrotic cell death, which plays a critical role in the formation of necrotic cores and plaque instability (Harrison et al., 2003).

The ubiquitin-proteasome system (UPS), which includes two sequential processes, namely ubiquitination and proteasome-mediated proteolysis, is the major non-lysosomal pathway for cellular proteolysis in eukaryotic cells (Hochstrasser, 1995; Ciechanover and Schwartz, 1998). The 26S proteasome complex is the proteolytic center of the UPS, which typically consists of three catalytic subunits, β1 (PSMB6), β2 (PSMB7), and β5 (PSMB5), that account for the caspase-like, trypsin-like, and chymotrypsin-like proteolytic activities, respectively (Hochstrasser, 1995; Ciechanover and Schwartz, 1998). In immune cells, and upon pro-inflammatory stimulation, these three subunits can be replaced by their inducible forms, namely β1i (PSMB9 and LMP2), β2i (PSMB10, LMP10, and Mecl-1), and β5i (PSMB8 and LMP7), to fill in a specialized type of proteasome called the immunoproteasome (Angeles et al., 2012; Basler et al., 2013). Compared to non-inducible constitutive proteasomes, immunoproteasomes elicit increased proteolytic capacity and greater efficiency in the presentation of major histocompatibility complex class I antigens to cytotoxic T lymphocytes (Angeles et al., 2012; Basler et al., 2013). Importantly, immunoproteasomes are also involved in other immune and non-immune activities, such as the activation of the nuclear factor kappa B (NF-κB) pathway and regulation of pro-inflammatory cytokine production, as well as management of oxidative stress and apoptosis (Angeles et al., 2012; Basler et al., 2013). Increased ubiquitin levels are observed in human systematic advanced plaques (Herrmann et al., 2002; Marfella et al., 2006; Versari et al., 2006), and pharmacological inhibition of ubiquitin activation attenuates experimental atherosclerosis (Liao et al., 2020b). Moreover, proteasome inhibition decreases early atherosclerosis (Feng et al., 2010; Wilck et al., 2012) but might promote advanced plaque instability (Van Herck et al., 2010; Wilck et al., 2017).

Recently, we uncovered a pro-atherogenic role of the immunoproteasome catalytic subunit LMP7 in apolipoprotein E (ApoE) knockout (ko) mice (Liao et al., 2020a). Here, we explored whether another subunit, LMP10, also contributes to diet-induced atherosclerosis.

## Materials and Methods

### Animals

LMP10 ko mice (C57BL/6 background) were purchased from Jackson Laboratory as described previously (Li et al., 2018) and crossed with ApoE ko mice (C57BL/6 background, Beijing Vital River Laboratory) to generate LMP10/ApoE double ko (dko) mice. All mice were maintained in specific-pathogen-free conditions on a 12-h light/12-h dark cycle with free access to water and diet. At 8–12 weeks of age, mice were subjected to an atherogenic diet (containing 0.5% cholesterol and 20% fat, Biotech HD) as previously described for 8 weeks to induce atherosclerosis (Liao et al., 2017). Only males were included in the experiments. All the animal procedures were approved by the Animal Care and Use Committee of Dalian Medical University and conformed to the United States National Institutes of Health Guide for the Care and Use of Laboratory Animals.

### Analysis of Plasma Lipids and Glucose

Blood samples were collected via retro-orbital puncture after a 4-h fasting. Plasma total cholesterol, triglycerides and glucose were measured with enzymatic kits (BioSino), according to the manufacturer’s guidance. For lipoprotein profiles, plasma samples were pooled from 4 to 5 mice per group and fractioned by fast protein liquid chromatography (FPLC) as we previously described (Liao et al., 2018). For glucose tolerance tests, mice were fasted for 4 h and then received an i.p. glucose (2 g/kg body weight) injection. Blood samples were collected before (time 0) and at 15, 30, 60, and 120 min after the glucose injection. Plasma glucose was measured as described above.

### Flow Cytometry

Flow cytometry analysis was performed as previously described (Liao et al., 2020b). Briefly, aorta samples were first cleaned of fatty tissues and digested with aorta dissociation enzyme stock solutions (125 U/ml collagenase type XI, 60 U/ml hyaluronidase type 1, 60 U/ml DNase I, and 450 U/ml collagenase type I, in 2.5 ml of PBS) to obtain single-cell suspensions. Then the single-cell suspensions were treated with Fc block and stained with CD45 PerCP-Cy5.5, CD11b FITC, F4/80 PE-Cy7, and CD206 APC. All the above antibodies were obtained from BD Biosciences. Events were acquired on a live gate on Fortessa flow cytometry (BD Biosciences).

### Bone Marrow Transplantation

Bone marrow (BM) transplantation was performed as previously described (Wang et al., 2016). Briefly, APOE ko mice of 8 weeks’ old were chosen as recipients and received lethal irradiation with 8.5 Gy dose from a cobalt source. BM was extracted by flushing the femurs and tibiae from donor mice (8 weeks of age) with RPMI-1640 (Gibco) supplemented with 2% fetal bovine serum and heparin (5 U/mL). Each recipient mouse was injected with 5 × 10^6^ BM cells by tail injection. Then the recipient mice were housed in clean, individually ventilated cages and fed with acidified, antibiotic water and sterilized food for 4 weeks to allow recovery.

### Atherosclerosis Analysis

Mice were sacrificed and flushed with PBS through the left ventricle. Atherosclerosis in the aorta and the aortic root were visualized and calculated as we previously described (Zhang et al., 2018). Briefly, the entire aortas were cleaned of the fatty tissues and cut open longitudinally under a dissecting microscope. Atherosclerosis in the *en face* aorta was visualized by Oil-red O (Sigma) staining and calculated as Oil-red O positive area percent entire inner surface area. The hearts were embedded in OCT (Sakura Finetek), snap-frozen in liquid nitrogen and cross-sectioned serially at 7 μm thick at the aortic root level. Cytosections were mounted from the point where all the 3 aortic valve cusps were clearly visible and 5 sections, each separated by 70 μm of the tissue, were included into 1 slide. Atherosclerosis in the aortic root was calculated as mean Oil-red O positive area averaged from 5 sections. Plaque macrophages and smooth muscle cells were visualized by immunohistochemical staining with anti-CD68 antibody (MCA1957, diluted at 1:300; Bio-Rad) and anti-SM22α antibody (ab14106, diluted at 1:300; Abcam), while collagen content and necrotic areas were visualized by Picro-Sirius Red staining and H&E staining, respectively. Lesional LMP10 expression was visualized by immunofluorescent co-staining using anti-LMP10 antibody (ab183506, diluted at 1: 200; Abcam) and anti-CD68 antibody (MCA1957, diluted at 1:300; Bio-Rad) or anti-SM22α anti-body (ab14106, diluted at 1:300; Abcam). All quantifications were performed with Image J software.

### Foam Cell Induction and Analysis

Murine peritoneal macrophages were induced by an intraperitoneal injection of 4% sterile thioglycollate media (Sigma) and harvested 72 h later. Collected macrophages were cultured in RPMI-1640 medium (Gibco) supplemented with 10% fetal bovine serum and 1% antibiotics (100 Units/ml penicillin and 100 μg/ml streptomycin) and maintained overnight in a humidified incubator at 37°C under 5% CO_2_. Then macrophages were treated with oxidized low density lipoprotein (ox-LDL) (50 μg/ml, Unionbiol) to induce foam cell formation. After 24 h of ox-LDL treatment, macrophages were washed, fixed with 4% paraformaldehyde solution for Oil-red O staining. To quantify intracellular cholesterol content, washed foam cells were treated with 0.5 ml hexane: isopropanol (3:2) and dried in fume hood. Intracellular total cholesterol level was measured by an Amplex Red Cholesterol Assay Kit (#A12216, Invitrogen) according to the manufacture’s protocol, normalized to the protein concentration determined by a bicinchoninic acid protein assay kit (Pierce Chemical).

### Western Blot Analysis

Protein samples were extracted using radioimmunoprecipitation assay (RIPA). The protein concentration of each extract was determined using a bicinchoninic acid protein assay kit (Pierce Chemical). Western blot analyses were performed using anti-LMP10 antibody (ab183506, Abcam), anti-phospho–IκBα (Ser32/36) (5A5, #9246, CST) and anti-IκBα (#9242, CST) antibodies, anti-phospho–NF-κB p65 (Ser536) (93H1, #3033, CST) and anti-NF-κB p65 (C22B4, #4764, CST) antibodies, with anti-GAPDH antibody (60004-1-lg, Proteintech) as control. Quantifications were performed with Image J software.

### Quantitative Real-Time PCR Analysis

RNA samples was extracted with Trizol (Invitrogen, United States). Complementary DNA was generated using a RT kit (MedChem Express). Quantitative real-time PCR was performed with SYBR Green qPCR reagents (MedChem Express), using primers listed in Table 1. All samples were quantitated using the comparative CT method and normalized to GAPDH.

**TABLE 1:** Primer sequences used in the quantitative real-time PCR.

**Name**	**Type**	**Sequence (5′–3′)**
MCP-1	Forward	TAAAAACCTGGATCGGAACCAAA
	Reverse	GCATTAGCTTCAGATTTACGGGT
IL-1β	Forward	CTTCCCCAGGGCATGTTAAG
	Reverse	ACCCTGAGCGACCTGTCTTG
IL-4	Forward	GGTCTCAACCCCCAGCTAGT
	Reverse	GCCGATGATCTCTCTCAAGTGAT
IL-6	Forward	TTCCATCCAGTTGCCTTCTTG
	Reverse	TTGGGAGTGGTATCCTCTGTGA
IL-10	Forward	GCTCTTACTGACTGGCATGAG
	Reverse	CGCAGCTCTAGGAGCATGTG
TGF-β	Forward	CCACCTGCAAGACCATCGAC
	Reverse	CTGGCGAGCCTTAGTTTGGAC
CD36	Forward	CTCGGATGGCTAGCTGATTACT
	Reverse	AGCACTTGCTTCTTGCCAAC
SR-A	Forward	AGGGAGTGGATAAATCAGTGCT
	Reverse	TCCTCCTGTTGCTTTGCTGT
ABCA1	Forward	CGACCATGAAAGTGACACGC
	Reverse	GACAGCTGGCAGGACAATCT
ABCG1	Forward	GGTTGCGACATTTGTGGGTC
	Reverse	GAAGATGGTCCTCAGGTGGC
SR-BI	Forward	GCCTCTGTTTCTCTCCCACC
	Reverse	CTGTCCGCTGAGAGAGTCCT
CD80	Forward	ACCCCCAACATAACTGAGTCT
	Reverse	TTCCAACCAAGAGAAGCGAGG
CD40	Forward	TGTCATCTGTGAAAAGGTGGTC
	Reverse	ACTGGAGCAGCGGTGTTATG
SOX3	Forward	GCCGACTGGAAACTGCTGA
	Reverse	CGTAGCGGTGCATCTGAGG
CD163	Forward	ATGGGTGGACACAGAATGGTT
	Reverse	CAGGAGCGTTAGTGACAGCAG
CD206	Forward	CTCTGTTCAGCTATTGGACGC
	Reverse	CGGAATTTCTGGGATTCAGCTTC
Arg1	Forward	CTCCAAGCCAAAGTCCTTAGAG
	Reverse	AGGAGCTGTCATTAGGGACATC
GAPDH	Forward	TGATGACATCAAGAAGGTGGTGAAG
	Reverse	TCCTTGGAGGCCATGTAGGCCAT

### Statistical Analysis

Data were analyzed with Prism software and presented as mean ± SEM. Normally distributed data was evaluated by unpaired *t*-test, while non-parametric data by Mann–Whitney test. A *P*-value < 0.05 was regarded as statistically significant.

## Results

### Upregulation of LMP10 in Lesional Macrophages in ApoE ko Mice

**FIGURE 1:**
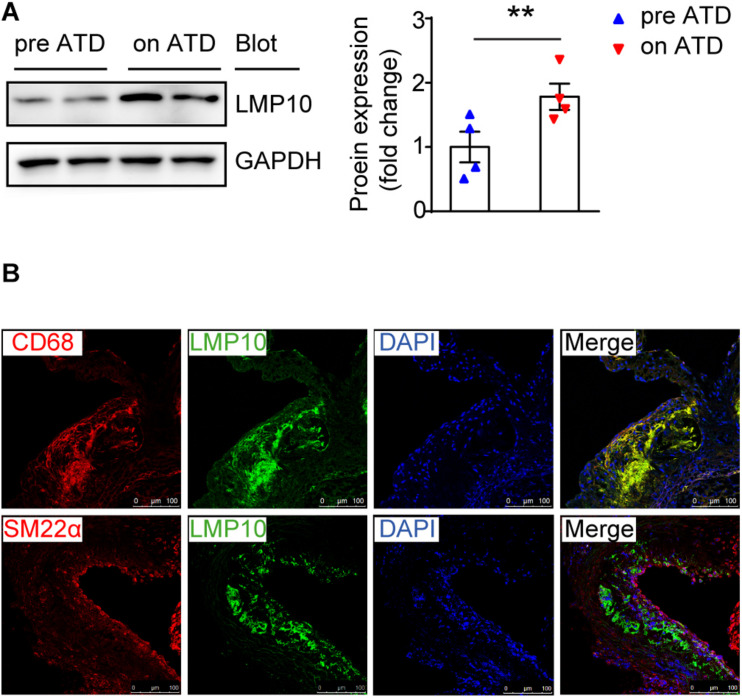
LMP10 was abundantly expressed in lesional macrophages in diet-induced atherosclerosis in ApoE ko mice. **(A)** Western-blot analysis and quantitation of aortic LMP10 protein expression in ApoE ko mice before and after 8 weeks of ATD feeding. *n* = 4 per group, **p < 0.01. **(B)** Immunofluorescent co-staining of LMP10 with CD68 or SM22α in the aortic root of ApoE ko mice before and after 8 weeks of ATD feeding.

To investigate the role of the immunoproteasome subunit LMP10 in the development of atherosclerosis, we first examined the expression of LMP10 in the aorta of ApoE ko mice after 8 weeks of atherogenic diet (ATD) feeding. Immunoblotting analysis showed that protein expression of LMP10 was significantly upregulated in the aorta of mice fed on an ATD, as compared to mice without ATD feeding (Figure 1A). To further identify which cell types in atherosclerotic lesions predominantly expressed LMP10, we co-stained LMP10 with macrophage biomarker CD68 or smooth muscle cell (SMC) biomarker SM22α. Immunofluorescent staining revealed that LMP10 was highly co-localized with CD68^+^ macrophages and hardly with SM22α^+^ SMCs (Figure 1B), indicating that increased LMP10 expression was mainly derived from lesional macrophages.

### Ablation of LMP10 Had no Effect on Plasma Metabolic Parameters in ApoE ko Mice After ATD Feeding

**FIGURE 2:**
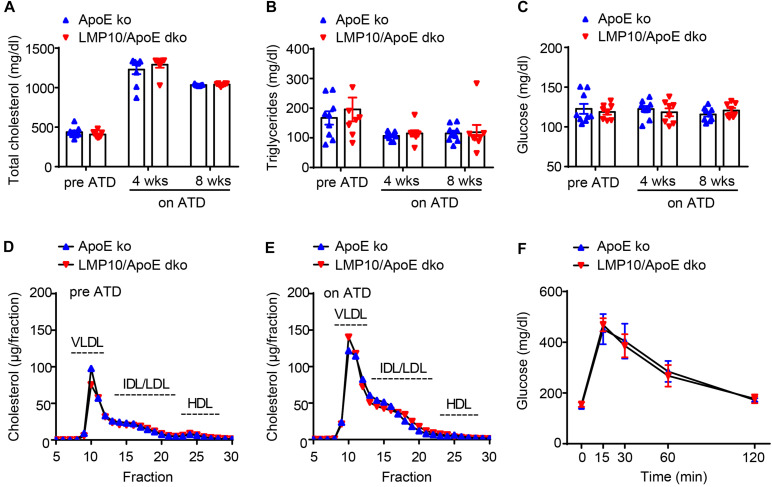
LMP10 deletion did not change plasma metabolic parameters in ApoE ko mice during ATD feeding. **(A–C)** Analysis of plasma total cholesterol **(A)**, triglycerides **(B)**, and glucose **(C)** levels before and after ATD feeding. **(D,E)** Analysis of plasma lipoprotein profiles by FPLC before **(D)** and after 8 weeks **(E)** of ATD feeding. **(F)** Analysis of glucose clearance by glucose tolerance test after 8 weeks on ATD. For **(A–C,F)**, *n* = 7–9 per group; for **(D,E)**, samples were pooled from 4 to 5 mice per group.

To identify whether LMP10 contributed to diet-induced atherogenesis, we generated LMP10 and ApoE double knockout (LMP10/ApoE dko) mice in addition to ApoE ko controls, which were fed the ATD for 8 weeks to induce atherogenesis. We first compared plasma metabolic parameters in mice during ATD feeding and found that total plasma cholesterol, triglycerides, and glucose levels were similar between the LMP10/ApoE dko mice and the ApoE ko controls, both before and after the ATD feeding (Figures 2A–C). Furthermore, there was no significant difference in the plasma lipoprotein profiles and the glucose clearance rate, detected by FPLC and glucose tolerance test, respectively, between the LMP10/ApoE dko and ApoE ko mice (Figures 2D–F), suggesting that LMP10 is not involved in the regulation of circulating lipids and glucose.

### Knockout of LMP10 Attenuated Diet-Induced Atherosclerosis in ApoE ko Mice

**FIGURE 3:**
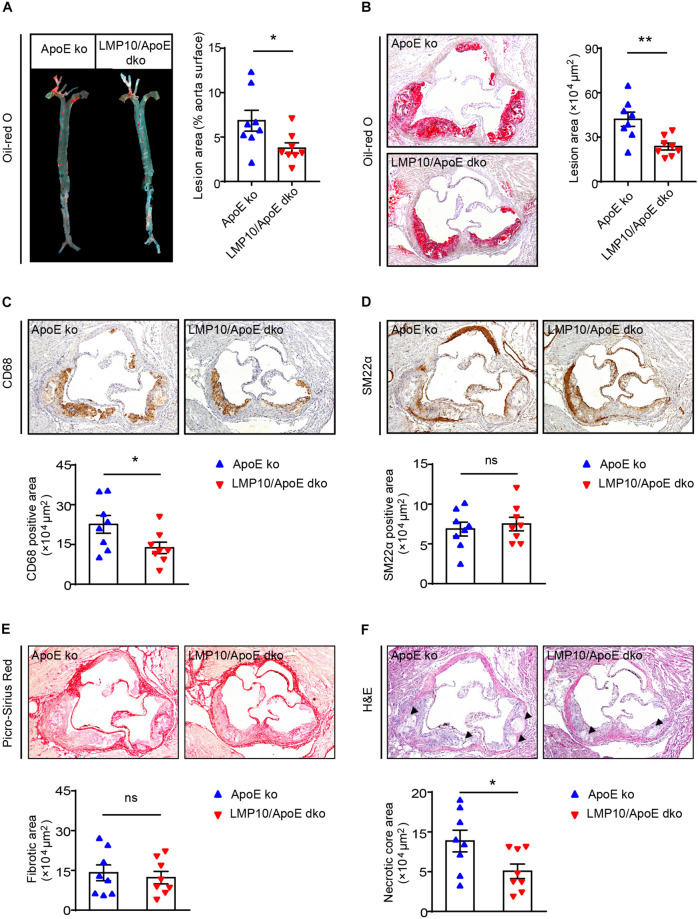
LMP10 deletion attenuated diet-induced atherosclerosis in ApoE ko mice. **(A)** Oil-red O staining and quantification of the atherosclerotic lesions in *en face* aortas. **(B)** Oil-red O staining and quantification of atherosclerotic lesions in the aortic root. **(C)** CD68 immunohistochemical staining and quantification of CD68^+^ macrophages in atherosclerotic lesions in the aortic root. **(D)** SM22α immunohistochemical staining and quantification of SM22α^+^ smooth muscle cells in atherosclerotic lesions in the aortic root. **(E)** Picro-Sirius Red staining and quantification of collagen in atherosclerotic lesions in the aortic root. **(F)** H&E staining and quantification of necrotic areas in atherosclerotic lesions in the aortic root. The triangle indicates the necrotic core. *n* = 8 per group, *p < 0.05; **p < 0.01; ns, not significant.

We then tested the effect of LMP10 on atherosclerotic plaques in the LMP10/ApoE dko and ApoE ko control mice after 8 weeks of ATD feeding. Oil-red O staining showed that atherosclerotic lesion areas on the entire inner aortic surface of LMP10/ApoE dko mice were markedly reduced, compared with that of the ApoE ko controls (Figure 3A). Reduction of lesion areas was further confirmed in the aortic root of LMP10/ApoE dko mice (Figure 3B).

Since macrophages play a key role in the onset and progression of atherosclerosis (Moore and Tabas, 2011; Tabas and Bornfeldt, 2016), we then examined the accumulation of macrophages in the aorta using anti-CD68 antibody. Immunochemical staining indicated that the infiltration of CD68^+^ macrophages in aortic plaques of LMP10/ApoE dko mice was significantly reduced compared to levels observed in ApoE ko controls (Figure 3C). Moreover, plaques of LMP10/ApoE dko mice had less necrotic core formation (Figure 3F). However, immunochemical staining with anti-SM22α antibody and Sirius Red staining showed no significant change in SM22α^+^SMCs or collagen content between LMP10/ApoE dko mice and ApoE ko control mice (Figures 3D,E). Together, these data suggest that knockout of LMP10 attenuates diet-induced atherosclerosis possibly through inhibition of macrophage infiltration and death in ApoE ko mice.

### Myeloid-Specific Knockout of LMP10 Inhibited Diet-Induced Atherosclerosis in ApoE ko Mice

**FIGURE 4:**
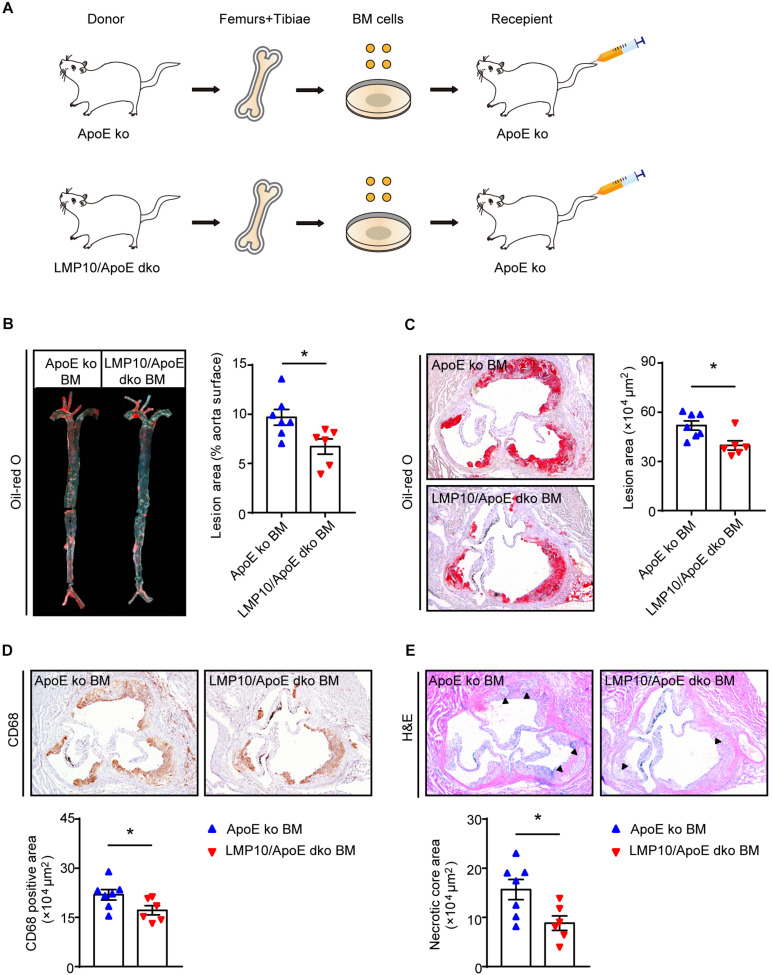
Macrophage-specific LMP10 deletion attenuated diet-induced atherosclerosis in ApoE ko mice. **(A)** Graphic representation of bone-marrow chimeric mice construction. **(B)** Oil-red O staining and quantification of atherosclerotic lesions in *en face* aortas. **(C)** Oil-red O staining and quantification of atherosclerotic lesions in the aortic root. **(D)** CD68 immunohistochemical staining and quantification of CD68^+^ cells in atherosclerotic lesions in the aortic root. **(E)** H&E staining and quantification of necrotic areas in atherosclerotic lesions in the aortic root. The triangle indicates the necrotic core. *n* = 6–7 per group, *p < 0.05.

Immunoproteasomes are known to be constitutively expressed in immune cells, particularly myeloid-derived monocytes and lymphocytes (Muchamuel et al., 2009). To directly explore whether myeloid-specific deletion of LMP10 affected diet-induced atherosclerosis, we created chimeric mice via bone marrow (BM) transplantation and treated them with ATD feeding for 8 weeks, after 4 weeks’ recovery from the transplantation. The procedure of chimeric mice construction was illustrated in Figure 4A, with LMP10/ApoE dko and ApoE ko mice as BM donors while ApoE ko mice as recipients. Our data showed that ApoE ko mice reconstituted with LMP10/ApoE dko BM cells exhibited a marked decrease in atherosclerotic lesion burdens in both the aorta and the aortic root (Figures 4B,C), as well as a marked decrease in macrophage infiltration, and necrotic core area in plaques (Figures 4D,E), as compared to ApoE ko mice reconstituted with ApoE ko BM cells. However, no significant change in plasma metabolic parameters was observed between the LMP10/ApoE dko and the ApoE ko control mice (data not shown). These results suggest that LMP10 in BM-derived inflammatory cells critically contributes to the formation of atherosclerosis in this model.

### Deletion of LMP10 Reduced Diet-Induced Macrophage Infiltration and Polarization in ApoE ko Mice

**FIGURE 5:**
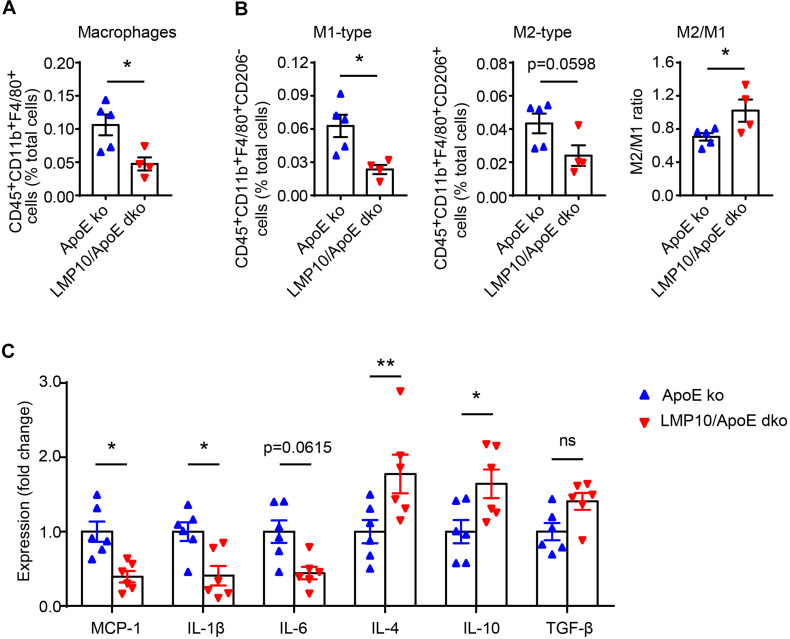
LMP10 deletion inhibited diet-induced aortic macrophage infiltration and polarization in ApoE ko mice. **(A,B)** Flow cytometry analysis of aortic total macrophage infiltration **(A)** and M1/M2-type macrophage numbers **(B)** after 8 weeks of ATD. *n* = 4–5 per group. **(C)** Real-time PCR analysis of aortic M1- and M2-associated inflammatory cytokine expression after 8 weeks of ATD. *n* = 6 per group, *p < 0.05; **p < 0.01; ns, not significant.

Macrophage infiltration and polarization play a vital role in the immune-inflammatory response during atherosclerosis (Ross, 1999). We therefore examined aortic macrophage infiltration and polarization after 8 weeks of ATD via flow cytometry. As shown in Figure 5A, the numbers of aortic F4/80^+^ macrophages were markedly lower in LMP10/ApoE dko mice when compared to ApoE ko controls. Consistent with this result, M1-type macrophages (marked as F4/80^+^CD206^–^ macrophages) and M2-type macrophages (marked as F4/80^+^CD206^+^ macrophages) were both decreased in LMP10/ApoE dko mice; however, the M2/M1 ratio was significantly increased, indicating a shift in polarization toward the M2 type (Figure 5B). Additionally, we analyzed changes in the expressions of M1 (such as MCP-1, IL-1β, and IL-6) and M2 (such as IL-4, IL-10, and TGF-β) signature genes in aortic lesions. Data from real-time PCR showed a roughly 50% decrease of M1-associated cytokine (MCP-1, IL-1β, and IL-6) expression and more than 1.5-fold increase of M2-associated cytokine (IL-4 and IL-10) expression in LMP10/ApoE dko mice compared with ApoE ko control mice (Figure 5C).

### LMP10 Deletion Blunted Macrophage Polarization and Inflammation via Inhibiting NF-κB Activation in ox-LDL-Treated Macrophages

**FIGURE 6:**
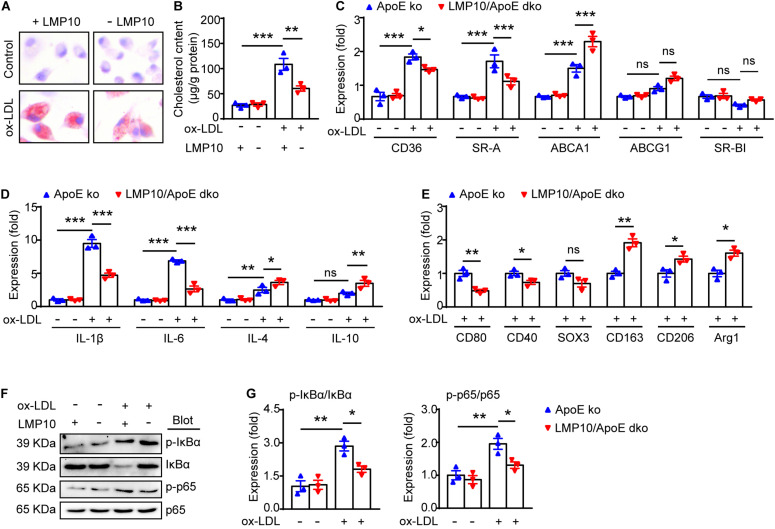
LMP10 deletion blunted macrophage polarization and inflammation via inhibiting NF-κB activation in ox-LDL-treated macrophages. **(A)** Oil-red O staining of isolated peritoneal macrophages treated with or without ox-LDL. **(B)** Quantitation of intracellular cholesterol content in isolated peritoneal macrophages treated with or without ox-LDL. **(C)** Real-time PCR analysis of cholesterol uptake- and efflux-associated gene expression in isolated peritoneal macrophages treated with or without ox-LDL. **(D)** Real-time PCR analysis of M1- and M2-associated inflammatory cytokine expression in isolated peritoneal macrophages treated with or without ox-LDL. **(E)** Real-time PCR analysis of M1/M2 signature gene expression in isolated peritoneal macrophages treated with ox-LDL. **(F,G)** Western-blot analysis **(F)** and quantitation **(G)** of IκBα phosphorylation and NF-κB activation in isolated peritoneal macrophages treated with or without ox-LDL. *n* = 3 per group, *p < 0.05; **p < 0.01; ***p < 0.001.

NF-κB signaling is a key transcriptional regulator for macrophage polarization and inflammation (Hoesel and Schmid, 2013; Lingappan, 2018). Therefore, we explored the NF-κB pathway in ox-LDL-treated macrophages isolated from the LMP10/ApoE dko mice and ApoE ko controls. Both Oil-red O staining and lipid extraction indicated that less cholesterol was accumulated in macrophages from LMP10/ApoE dko mice after ox-LDL stimulation (Figures 6A,B). Further, using real-time PCR analysis, we found in macrophages from LMP10/ApoE dko mice that genes associated with cholesterol uptake (CD36 and SR-A) were decreased, while those with cholesterol efflux (ABCA1) increased (Figure 6C); in addition, expressions of M1-associated cytokines (IL-1β and IL-6) and M1 signature genes (such as CD80, CD40 and SOX3, etc.) were both inhibited, while those of M2-associated cytokines (IL-4 and IL-10) and M2 signature genes (such as CD163, CD206, and Arg1, etc.) upregulated (Figures 6D,E). Finally, western blotting showed that the phosphorylation of IκBα, the inhibitor of NF-κB, and the phosphorylation/activation of NF-κB p65 were all reduced in macrophages from LMP10/ApoE dko mice challenged with ox-LDL, which might contribute to decreased polarization toward M1 and lower inflammation observed in LMP10 deleted conditions (Figures 6F,G).

## Discussion

**FIGURE 7:**
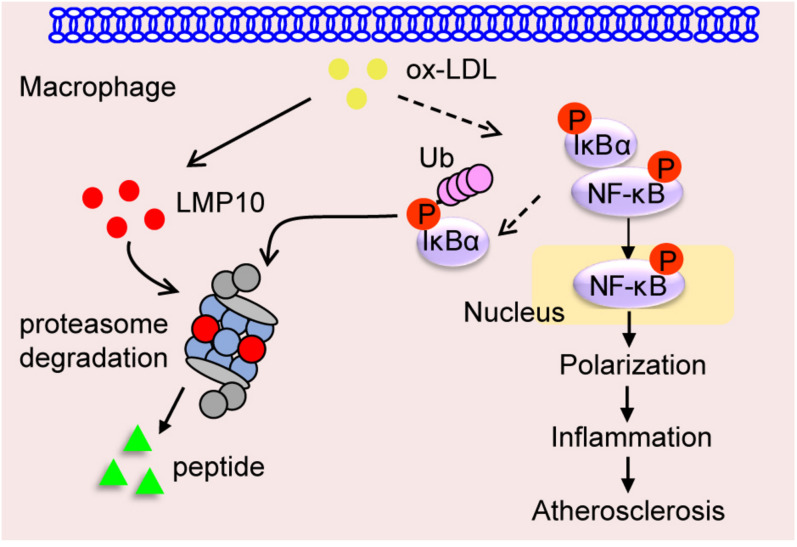
Graphic representation of macrophage LMP10 in diet-induced atherosclerosis. The accumulation of lipids (such as ox-LDL) induces macrophage LMP10 expression, which facilitates the degradation of phosphorylated IκBα, followed by activation and translocation of phosphorylated NF-κB p65, finally promoting macrophage polarization and inflammation, therefore contributing to atherosclerosis.

Immunoproteasomes have been implicated in human atherosclerosis (Herrmann et al., 2012). Previous research has shown that LMP7 expression is increased in the shoulder areas of symptomatic carotid plaques and in correlation with inflammatory cell infiltration, but the causative roles and potential mechanisms of immunoproteasomes in atherosclerosis remain unclear (Herrmann et al., 2012). Recently, we demonstrated in ApoE ko mice that ATD feeding significantly induced LMP7 expression in plaque macrophages, and that genetic and pharmaceutical inhibition of LMP7 attenuated diet-induced atherosclerosis (Liao et al., 2020a). Furthermore, we identified in isolated macrophages that LMP7-mediated regulation of atherosclerosis was associated with efferocytosis of apoptotic cells during foam cell formation (Liao et al., 2020a). In the current study, we explored whether another immunoproteasome catalytic subunit LMP10 contributed to experimental atherosclerosis in ApoE ko mice. We demonstrated that (1) LMP10 is highly expressed in lesional macrophages in diet-induced atherosclerosis; (2) LMP10 deletion, especially myeloid-specific LMP10 deletion, attenuates diet-induced atherosclerosis; and (3) LMP10 deletion might inhibit NF-κB-mediated macrophage polarization and inflammation. The working model is illustrated in Figure 7. Interestingly, our current and previous studies all indicate that deletion of immunoproteasome subunit, either LMP10 or LMP7, do not elicit profound influence on plasma metabolic parameters in ApoE ko mice, suggesting that immunoproteasomes are possibly not involved in the regulation of global lipid/glucose metabolism during diet-induced atherosclerosis.

The roles of immunoproteasomes in cardiovascular health and disease have been extensively explored recently. In a mouse model of angiotensin II (Ang II)-induced cardiac remodeling, proteolytic activities and expression of the immunoproteasome subunits LMP2, LMP10, and LMP7 were found to be significantly up-regulated (Li et al., 2015). Genetic and pharmaceutical inactivation of LMP7 or LMP10 subunits is sufficient to elicit profound impacts on both ventricular hypertrophy and atrial fibrillation induced by Ang II infusion (Li et al., 2018, 2019; Xie et al., 2019, 2020). In addition to pressure overload, immunoproteasomes are also involved in other cardiac diseases, such as doxorubicin-induced cardiotoxicity (Zhao et al., 2015) and deoxycorticosterone acetate (DOCA)/Salt-induced heart failure (Yan et al., 2017). It was demonstrated that doxorubicin significantly decreases proteolysis activities and immunoproteasome (LMP2, LMP10, and LMP7) expression. Overexpression of immunoproteasome catalytic subunits (LMP2, LMP10, or LMP7) protected against doxorubicin-induced cardiomyocyte apoptosis, while inactivation promoted apoptosis (Zhao et al., 2015). In DOCA/salt-induced cardiac hypertrophy, LMP10 subunit expression was found to be significantly increased, while inactivation of LMP10 ameliorated DOCA/salt-induced cardiac fibrosis and inflammation (Yan et al., 2017). The present study and our previous LMP7 data in diet-induced atherosclerosis, together with the above described evidence, expand our knowledge about the role of immunoproteasomes in cardiovascular homeostasis and, most importantly, provide new therapeutic targets for human cardiovascular diseases.

Macrophages during inflammation are particularly dynamic, with diverse functional and phenotypic heterogeneity, depending on microenvironment and activated intracellular signaling pathways (Tabas and Bornfeldt, 2016). The M1/M2 nomenclature is well recognized, with M1-type macrophages tending to secrete pro-inflammatory cytokines (such as MCP-1, IL-1β, and IL-6) to augment the inflammatory cascade, while M2 cells tend to secrete anti-inflammatory cytokines (such as IL-4, IL-10, and TGF-β) helping to resolve inflammation. Previous studies have suggested a potential role of immunoproteasomes in macrophage polarization toward M1/M2 phenotypes. For example, in diet-induced obesity, LMP7 ablation promoted adipose macrophages to switch toward the anti-inflammatory M2 phenotype (Kimura et al., 2015b). Similarly, data from alveolar macrophages showed that LMP7 inactivation increased M2 polarization triggered by IL-4, but had no effect on LPS/IFN-γ-triggered M1 polarization (Chen et al., 2016). However, whether immunoproteasomes contribute to plaque macrophage polarization during atherosclerosis has not been elucidated. In the present study, we discussed the diet-induced M1/M2 polarization in atherosclerosis when LMP10 was inactivated. We observed an increased M2/M1 ratio in the vessels after ATD feeding in LMP10/ApoE dko mice, although the numbers of both M2- and M1-type macrophages were decreased, compared to ApoE ko controls (Figure 5B). Similarly, the expression of M1-associated cytokines was decreased, while the expression of M2-associated cytokines was increased in LMP10/ApoE dko mice (Figure 5C). These data suggested that LMP10 deletion reduced macrophage polarization and inflammation, which was further confirmed in ox-LDL-induced foam cell models, using primary peritoneal macrophages collected from LMP10/ApoE dko mice and ApoE ko controls. Our data, for the first time, demonstrates that the immunoproteasome LMP10 subunit may be able to regulate macrophage polarization and inflammation, therefore contributing to diet-induced atherosclerosis.

Plaque macrophage polarization toward M1/M2 type is known to be regulated by several transcriptional signaling pathways. The key transcriptional factors for these signaling pathways include NF-κB, signal transducer and activator of transcription (STAT), peroxisome proliferator-activated receptor-γ (PPARγ), cAMP responsive element-binding protein (CREB), and interferon regulatory factors (IRFs) (De Paoli et al., 2014; Wang et al., 2014). Of these transcriptional pathways, NF-κB signaling has been suggested as one of the major regulatory mechanisms of immunoproteasomes in immune-inflammatory responses in cardiovascular diseases (Yang et al., 2009; Angeles et al., 2012; Kimura et al., 2015a). For example, in Ang II-induced cardiac hypertrophy, activation of immunoproteasomes was found to promote degradation of MKP-1 and IκBα and subsequent activation of STAT1 and NF-κB, thereby leading to Th1 cell differentiation and cardiac remodeling (Qin et al., 2018); while in DOCA/salt-induced cardiac hypertrophy, the immunoproteasome LMP10 subunit was shown to activate IκBα/ NF-κB and TGF-β1/Smad2/3 signaling to facilitate cardiac fibrosis and inflammation (Yan et al., 2017). The regulatory effects of LMP10 on IκBα/NF-κB activation and signaling has further been confirmed in hypertensive atrial fibrillation and retinopathy (Li et al., 2018; Wang et al., 2018). In these two models, LMP10 promoted PTEN degradation and subsequent AKT1 activation, which then stimulated IKKβ-mediated IκBα phosphorylation and degradation, ultimately facilitating activation of NF-κB pathway (Li et al., 2018; Wang et al., 2018). Importantly, blocking NF-κB activation by administration of IKKβ specific inhibitor IMD-0354 remarkably blunted inflammation and disease phenotypes (Li et al., 2018; Wang et al., 2018). These studies indicated that LMP10 targets NF-κB activation possibly through PTEN/AKT/IKK signaling. Interestingly, our recent study on experimental atherosclerosis also supports LMP7-mediated transcriptional regulation of efferocytosis through oxidized LDL-induced IκBα degradation and subsequent NF-κB activation (Liao et al., 2020a). In the current study, we observed that oxidized LDL-induced degradation of IκBα and activation of NF-κB pathway was also reduced in LMP10-deleted macrophages (Figures 6F,G), suggesting that LMP10 deletion inhibited NF-κB activation partially through blocking IκBα degradation.

## Conclusion

Our data demonstrated for the first time that the immunoproteasome subunit LMP10 promoted diet-induced atherosclerosis in ApoE ko mice partially through regulation of NF-κB-mediated macrophage polarization and inflammation. Therefore, LMP10 might be exploited as a potential pharmaceutical target for atherosclerosis. Further studies might be needed to examine the involvement of other signaling pathways such as STAT, PPARγ, CREB, and IRFs in macrophage polarization and inflammation, as well as other mechanisms, such as oxidative stress and autophagy, in LMP10-mediated atherosclerosis in this model.

## Data Availability Statement

The original contributions presented in the study are included in the article/supplementary material, further inquiries can be directed to the corresponding author/s.

## Ethics Statement

The animal study was reviewed and approved by the Animal Care and Use Committee of Dalian Medical University.

## Author Contributions

JL, Y-LX, and H-HL conceived the project and designed the experiments. JL, XA, XY, Q-YL, SL, YX, and JB performed the experiments and acquired the data. JL analyzed the data and wrote the original draft of the manuscript. Y-LX and H-HL reviewed and edited the manuscript. All authors have read and approved the final manuscript.

## Conflict of Interest

The authors declare that the research was conducted in the absence of any commercial or financial relationships that could be construed as a potential conflict of interest.

## Abbreviations

UPS: ubiquitin-proteasome system
ApoE: apolipoprotein E
ko: knockout
dko: double knockout
ATD: atherogenic diet
FPLC: fast protein liquid chromatography
BM: bone marrow
ox-LDL: oxidized low density lipoprotein
NF- κ B: nuclear factor kappa B
I κ B: inhibitor of NF- κ B
Ang II: angiotensin II
SMC: smooth muscle cell
DOCA: deoxycorticosterone acetate.
